# Supplementary material for: Population-Based Registry Analysis of Antidiabetics Dispensations: Trend Use in Spain between 2015 and 2018 with Reference to Driving
Source: Pharmaceuticals (Basel). 2020 Jul 25;13(8):165. doi: 10.3390/ph13080165 (PMC7464462; doi:10.3390/ph13080165)
Supplement: Supplementary file 1 [file pharmaceuticals-13-00165-s001.pdf]

Table S1. Oral antidiabetics and insulins available in Castile and León (2015–2018).

Type of antidiabetic	Code ATC	Name	Pictogram	DRUID category
Insulins	A10AB01	Insulin (human), fast acting	√	I
	A10AB04	Insulin lispro, fast acting	√	I
	A10AB05	Insulin aspart, fast acting	√	I
	A10AB06	Insulin glulisine	√	I
	A10AC01	Insulin (human), intermediate acting	√	I
	A10AC04	Insulin lispro, intermediate acting	√	I
	A10AD01	Insulin (human), intermidiate or long acting combined with fast acting	√	I
	A10AD04	Insulin lispro, intermidiate or long acting combined with fast acting	√	I
	A10AD05	Insulin aspart, intermidiate or long acting combined with fast acting	√	I
	A10AE04	Insuline glargine	√	I
	A10AE05	Insulin detemir	√	I
	A10AE06	Insulin degludec (available from 2016)	√	I
Oral antidiabetics	A10BA02	Metformin	X	0
	A10BB01	Glibenclamide	√	I
	A10BB07	Glipizide	√	I
	A10BB09	Glicazide	√	I
	A10BB12	Glimepiride	√	I
	A10BB91	Glisentide	√	I
	A10BD05	Metformin and pioglitazone	X	0
	A10BD06	Glimepiride and pioglitazone	√	I
	A10BD07	Metformin and sitagliptin	√	I
	A10BD08	Metformin and vildagliptin	√	I
	A10BD09	Pioglitazone and alogliptin	√	I
	A10BD10	Metformine and saxagliptin	√	I
	A10BD11	Metformin and linagliptin	√	I
	A10BD13	Metformin and alogliptin	√	I
	A10BD15	Metformin and dapagliflozin	√	I
	A10BD16	Metformin and canagliflozin	√	I
	A10BD19	Linagliptin and empagliflozin (available from 2018)	√	I
	A10BD20	Metformin and empagliflozin (available from 2016)	√	I
	A10BF01	Acarbose	X	0
	A10BF02	Miglitol	X	0
	A10BG03	Pioglitazone	X	0
	A10BH01	Sitagliptin	√	I
	A10BH02	Vildagliptin	√	I
	A10BH03	Saxagliptin	√	I
	A10BH04	Alogliptin	√	I
	A10BH05	Linagliptin	√	I
	A10BJ01	Exenatide	√	I
	A10BJ02	Liraglutide	√	I
	A10BJ03	Lixisenatide	√	I
	A10BJ04	Albiglutide	√	I
	A10BJ05	Dulaglutide	√	I
	A10BK01	Dapagliflozin	√	I
	A10BK02	Canagliflozin	√	I
	A10BK03	Empagliflozin	√	I
	A10BX01	Guar gum	X	0
	A10BX02	Repaglinide	√	I
	A10BX03	Nateglinide	√	I

ATC, DRUID, DRiving Under the Influence of Drugs, alcohol, and medicines.

Table S2. Evolution of the Castile and León population and drivers´s licences (2015–2018).

Population												
**Rank age**	**2015**			**2016**			**2017**			**2018**		
	**Men**	**Women**	**Total**	**Men**	**Women**	**Total**	**Men**	**Women**	**Total**	**Men**	**Women**	**Total**
0-4	45 405	42 504	87 909	44 382	41 386	85 768	42 905	40 144	83 049	41 604	39 121	80 725
5-9	50 925	48 078	99 003	50 665	47 821	98 486	50 035	47 344	97 379	48 594	45 657	94 251
10-14	49 439	47 220	96 659	49 847	47 730	97 577	50 316	48 259	98 575	51 124	48 407	99 531
15-19	48 620	46 904	95 524	48 862	46 935	95 797	48 939	46 706	95 645	49 610	47 885	97 495
20-24	54 724	53 382	108 106	53 230	52 333	105 563	52 182	51 246	103 428	51 428	50 777	102 205
25-29	62 787	61 247	124 034	61 109	59 382	120 491	59 522	57 531	117 053	58 298	56 506	114 804
30-34	75 089	71 664	146 753	71 742	68 841	140 583	68 575	66 051	134 626	65 942	63 241	129 183
35-39	90 372	87 031	177 403	87 267	83 676	170 943	83 600	80 400	164 000	79 663	76 944	156 607
40-44	92 686	89 879	182 565	92 967	90 094	183 061	92 799	89 681	182 480	92 434	89 499	181 933
45-49	93 082	91 643	184 725	93 035	91 392	184 427	92 076	90 588	182 664	91 744	89 952	181 696
50-54	93 252	90 618	183 870	93 251	91 395	184 646	93 500	91 893	185 393	92 913	92 426	185 339
55-59	87 280	84 212	171 492	88 988	85 894	174 882	89 831	86 956	176 787	90 852	87 988	178 840
60-64	72 448	69 337	141 785	75 073	72 029	147 102	77 520	74 875	152 395	79 640	77 583	157 223
65-69	65 430	66 777	132 207	66 403	67 268	133 671	67 615	68 053	135 668	67 660	67 825	135 485
70-74	56 526	61 968	118 494	58 076	63 396	121 472	58 913	64 067	122 980	60 320	64 908	125 228
75-79	45 154	56 939	102 093	43 540	53 807	97 347	43 510	52 990	96 500	46 205	55 251	101 456
80-84	44 543	62 354	106 897	44 319	62 772	107 091	42 312	60 175	102 487	39 465	56 065	95 530
85-89	27 547	46 335	73 882	28 618	47 555	76 173	29 407	48 337	77 744	29 731	48 690	78 421
≥ 90	13 282	30 034	43 316	14 119	31 809	45 928	14 662	32 970	47 632	15 333	34 407	49 740
**Total**	**1 168 591**	**1 208 126**	**2 376 717**	**1 165 493**	**1 205 515**	**2 371 008**	**1 158 219**	**1 198 266**	**2 356 485**	**1 152 560**	**1 193 132**	**2 345 692**

Licensed drivers												
**Rank age**	**2015**			**2016**			**2017**			**2018**		
	**Men**	**Women**	**Total**	**Men**	**Women**	**Total**	**Men**	**Women**	**Total**	**Men**	**Women**	**Total**
15-19	9 282	5 586	14 868	9 238	5 634	14 872	8 357	4 689	13 046	8 702	5 102	13 804
20-24	43 294	35 387	78 681	42 165	34 280	76 445	40 859	33 207	74 066	39 837	32 570	72 407
25-29	55 831	50 618	106 449	53 617	48 755	102 372	51 913	46 861	98 774	50 269	45 329	95 598
30-34	69 810	61 387	131 197	66 192	58 489	124 681	62 677	56 134	118 811	59 765	53 560	113 325
35-39	86 841	75 838	162 679	83 112	72 880	155 992	79 204	70 129	149 333	74 693	66 771	141 464

40-44	89 294	76 277	165 571	88 673	76 856	165 529	88 025	76 717	164 742	87 163	76 513	163 676
45-49	90 151	74 310	164 461	89 641	74 389	164 030	88 611	74 242	162 853	87 961	74 534	162 495
50-54	90 450	67 282	157 732	90 290	69 036	159 326	90 158	70 695	160 853	89 420	72 062	161 482
55-59	85 820	56 346	142 166	87 607	59 781	147 388	88 543	62 443	150 986	89 826	64 796	154 622
60-64	71 450	36 255	107 705	74 219	40 173	114 392	76 748	44 338	121 086	79 194	48 281	127 475
65-69	62 572	23 964	86 536	63 824	26 068	89 892	65 577	28 466	94 043	65 978	30 748	96 726
70-74	51 161	12 390	63 551	52 595	13 755	66 350	53 663	15 096	68 759	55 094	16 442	71 536
75-79	35 993	5 000	40 993	34 852	5 277	40 129	35 960	6 008	41 968	38 829	7 105	45 934
80-84	28 304	1 941	30 245	28 194	2 055	30 249	27 809	2 319	30 128	26 500	2 491	28 991
85-89	14 160	429	14 589	14 133	484	14 617	14 548	588	15 136	14 814	661	15 475
≥ 90	2 944	22	2 966	5 669	50	5 719	6 176	77	6 253	7 350	112	7 462
Total	887 357	583 032	1 470 389	884 021	587 962	1 471 983	878 828	592 009	1 470 837	875 395	597 077	1 472 472

Table S3. List of the 20 antidiabetics with the pictogram "medicines and driving" more consumed in Castile and León into the study period (packages/year).

Code ATC	Name	Antidiabetic Type	Packages/year		
			Men	Women	Total
A10BD07	Metformin and sitagliptin	Oral antidiabetic	107757	73555	181311
A10BD08	Metformin and vildagliptin	Oral antidiabetic	90540	64416	154956
A10AE04	Insuline glargine	Insulin	81440	68007	149446
A10BB09	Glicazide	Oral antidiabetic	46076	41839	87915
A10BX02	Repaglinide	Oral antidiabetic	39302	36674	75976
A10BH01	Sitagliptin	Oral antidiabetic	28064	33067	61131
A10BH05	Linagliptin	Oral antidiabetic	26546	27613	54159
A10BB12	Glimepiride	Oral antidiabetic	24021	20851	44872
A10AD05	Insulin aspart, intermidiate or long acting combined with fast acting	Insulin	16333	16731	33064
A10AE05	Insulin detemir	Insulin	16077	16479	32556
A10AB05	Insulin aspart, fast acting	Insulin	17641	13987	31628
A10BK01	Dapagliflozin	Oral antidiabetic	16602	12370	28972
A10BH02	Vildagliptin	Oral antidiabetic	12891	16047	28938
A10BD11	Metformin and linagliptin	Oral antidiabetic	15908	11895	27803
A10BJ02	Liraglutide	Oral antidiabetic	13435	12447	25882
A10AB04	Insulin lispro, fast acting	Insulin	11148	9867	21015
A10AD04	Insulin lispro, intermidiate or long acting combined with fast acting	Insulin	99983	10002	20000
A10BJ05	Dulaglutide	Oral antidiabetic	10758	8652	19410
A10BK03	Empagliflozin	Oral antidiabetic	11649	76238	19272
A10BD15	Metformin and dapagliflozin	Oral antidiabetic	9976	6917	16893

Table S4. Consumption of oral antidiabetic without the pictogram "medicines and driving" in Castile and León into the study period (packages/year).

Code ATC	Name	Packages/year		
		**Men**	**Women**	**Total**
A10BA02	Metformin	397527	318359	715886
A10BF01	Acarbose	3983	4091	8073
A10BG03	Pioglitazone	2459	2463	4922
A10BD05	Metfomin and pioglitazone	2601	1690	4291
A10BX01	Guar gum	166	271	437
